# Weight No Advantage in Artificial Lower Dentures

**Published:** 1858-07

**Authors:** W. S. Putnam

**Affiliations:** New York.


### ARTICLE IV.

### *Weight no advantage in Artificial Lower Dentures.*

To the Editors of the Journal:

I am cautioned by friends not to advocate such a theory as the above, if I would avoid the ridicule of the profession at large. But I have no *theory* to offer, beyond what may be contained in the attempted solution of an ascertained *fact*, and therefore present my views, confident that those of the profession whose opinions I value, will respect my statements, however much they may differ from my explanation.

I make the negative assertion—broad as it may seem—that weight is, in *no case*, an advantage in a lower piece. I still further make the positive assertion, that the lighter a lower denture can be made, (consistently of course with strength and a proper extent of bearing surface,) the greater will be its utility and comfort to the patient.

These conclusions I draw from my observations of over sixty cases, in which lower dentures of given weight have been substituted for others of from $\frac{1}{2}$ to $\frac{1}{4}$ the weight, yet having equal or greater bulk and bearing surface.

In twelve of these cases, (all in the city of New York, and to which reference is permitted,) where the ridge was absorbed on a level with the muscles, and could be traced with the finger but not by the eye, the difficulty was mainly to keep the lower denture in place while eating and talking, a difficulty overcome to the satisfaction of all concerned, upon the substitution of lighter pieces having equal or greater width; yet, both heavy and light pieces were made with equal care and from equally good impressions.

I have given the *fact* in the case, so far as *my* experience goes. Let any one who wishes to oppose fact to fact, be careful that the conditions of the several cases are equal.

To urge the greater comfort of a heavy piece, with thick rounded edges, over a lighter one with thin cutting edges, or the greater utility of a piece having a broad bearing, over one that rests on a narrow ridge, is manifestly unfair.

The rounded edges and the broad bearing, give to certain styles of work, advantages which have, without due consideration, been assigned to weight—advantages so decided as to completely mask the *dis*advantage of weight, until brought to light in the use of a style, which while admitting, in the most perfect manner, narrowness of edge and breadth of bearing, can, in point of lightness, have no rival, until aluminium comes to be pressed into the service of the dentist. Until these opposing facts, the result of fairly tried experiments, shall show to the contrary, I state as a *truth*, against which my own experience offers no single instance, that weight in lower dentures—other things equal—is a *disadvantage*.

Now for my theory, which offering with diffidence, I am ready to yield for one more satisfactory.

Every one familiar with the power of muscular action, must admit that mere weight within the limits allowed to a dental plate, is inadequate to prevent a ready displacement.

In all ordinary cases, the plate is so trimmed as not to come in range of their action—just those cases in which the advocates of weight do not claim its necessity or advantage. But in other cases, where there is apparently no ridge, any form of plate will rest more or less upon the muscles, no matter how much trimming may have been given it. Why then will the heaviest plates made for such cases—2 to 4 ozs.—be "afloat" or "all about the mouth," as some express it, irritating first in one place and then in another?

We answer, because of the irritation of *constant* pressure. A piece of less weight, will press equally as much in the act of mastication, but at other times more lightly, producing less soreness of gum and less weariness of muscle

In all cases of irritation by heavy or light plates, the involuntary movement of muscles to displace them, is as natural as to wink the eye, to displace a fly from any point near it; and further, that no possible amount of weight can be endured upon any muscle in the mouth, to prevent that involuntary action throwing the plate as high as it would the same bulk in cork.

We need no longer disguise the fact, that the vulcanite base, is *the* base and proof of these arguments, as well as their demonstration; and while that style of artificial dentistry is open for criticism, it would be well, perhaps, for each "dentist of high standing" to investigate a little farther, so as not to mistake it for worn out temporary work on gutta percha "considerably disintegrated."

W. S. PUTNAM,
*New York.*

